# Supplementary material for: A stress-induced source of phonon bursts and quasiparticle poisoning
Source: Nat Commun. 2024 Jul 31;15:6444. doi: 10.1038/s41467-024-50173-8 (PMC11292027; doi:10.1038/s41467-024-50173-8)
Supplement: Supplementary file 1 — Supplementary Information [file 41467_2024_50173_MOESM1_ESM.pdf]

A Stress Induced Source of Phonon Bursts and Quasiparticle Poisoning: Supplementary Information

R. Anthony-Petersen,[1] A. Biekert,[1,2] R. Bunker,[3] C.L. Chang,[4,5,6] Y.-Y. Chang,[1] L. Chaplinsky,[7] E. Fascione,[8,9] C.W. Fink,[1] M. Garcia-Sciveres,[2] R. Germond,[8,9] W. Guo,[10,11] S.A. Hertel,[7] Z. Hong,[12] N.A. Kurinsky,[13] X. Li,[2] J. Lin,[1,2] M. Lisovenko,[4] R. Mahapatra,[14] A.J. Mayer,[9] D.N. McKinsey,[1,2] S. Mehrotra,[1] N. Mirabolfathi,[14] B. Neblosky,[15] W.A. Page,[1,*] P.K. Patel,[7] B. Penning,[16,17] H.D. Pinckney,[7] M. Platt,[14] M. Pyle,[1] M. Reed,[1] R.K. Romani,[1,*] H. Santana Queiroz,[1] B. Sadoulet,[1] B. Serfass,[1] R. Smith,[1,2] P. Sorensen,[2] B. Suerfu,[1,2,18] A. Suzuki,[2] R. Underwood,[8] V. Velan,[1,2] G. Wang,[4] Y. Wang,[1,2] S.L. Watkins,[1] M.R. Williams,[16] V. Yefremenko,[4] and J. Zhang[4]

[1] *Department of Physics, University of California, Berkeley, CA 94720, USA*
[2] *Lawrence Berkeley National Laboratory, Berkeley, CA 94720, USA*
[3] *Pacific Northwest National Laboratory, Richland, WA 99352, USA*
[4] *High Energy Physics Division, Argonne National Laboratory, 9700 S. Cass Avenue, Argonne, Illinois 60439, USA*
[5] *Department of Astronomy and Astrophysics, University of Chicago, 5640 South Ellis Avenue, Chicago, Illinois 60637, USA*
[6] *Kavli Institute for Cosmological Physics, University of Chicago, 5640 South Ellis Avenue, Chicago, Illinois 60637, USA*
[7] *Department of Physics, University of Massachusetts, Amherst, Massachusetts 01003, USA*
[8] *Department of Physics, Queen's University, Kingston, ON K7L 3N6, Canada*
[9] *TRIUMF, Vancouver, BC V6T 2A3, Canada*
[10] *Mechanical Engineering Department, FAMU-FSU College of Engineering, Florida State University, Tallahassee, Florida 32310, USA*
[11] *National High Magnetic Field Laboratory, 1800 East Paul Dirac Drive, Tallahassee, Florida 32310, USA*
[12] *Department of Physics, University of Toronto, Toronto, ON M5S 1A7, Canada*
[13] *SLAC National Accelerator Laboratory/Kavli Institute for Particle Astrophysics and Cosmology, Menlo Park, California 94025, USA*
[14] *Department of Physics and Astronomy, and the Mitchell Institute for Fundamental Physics and Astronomy, Texas A&M University, College Station, Texas 77843, USA*
[15] *Department of Physics and Astronomy, Northwestern University, Evanston, IL 60208, USA*
[16] *University of Michigan, Randall Laboratory of Physics, Ann Arbor, MI 48109, USA*
[17] *Physik-Institut, University of Zürich, Zürich, Switzerland*
[18] *International Center for Quantum-field Measurement Systems for Studies of the Universe and Particles (QUP, WPI), High Energy Accelerator Research Organization (KEK), Tsukuba, 305-0801, Ibaraki, Japan*

SUPPLEMENTARY NOTE 1: COMPARISON WITH OTHER CALORIMETRIC EXPERIMENTS

For comparison, we show the low-energy spectra measured with our HS and LS devices alongside the spectra observed in the CPD [1] and CRESST-III [2] calorimeters in Supplementary Fig. 1. This comparison assumes a phonon collection efficiency of 25% in the HS and LS calorimeters, consistent with the observed collection efficiencies for devices of this type within $\mathcal{O}(2)$ [1]. When normalizing by detector mass, the background rates in our LS and HS detectors are significantly above previously above rates reported for the CPD and CRESST-III detectors.

However, scaling by sensor volume (taken here as the aluminium volume) may be more appropriate if backgrounds originate in the sensor films (as hypothesized in Section 2.1 of the main text) rather than the bulk of the detector. The center subfigure of Fig. S1 attempts to make this correction, assuming that the CRESST-III phonon sensors are 1 um thick and with the dimensions described in Ref. [3].

Given that the low energy background rate has been observed to decrease with time both in CRESST detectors [4, 5] and in the detectors in this work, corrections for the time since the detector was initially cooled to base temperature are necessary for any effort to compare background rates on an equal footing. For the CPD detector, we assume that the rate decreases similarly to the rate in the LS detector in the 38-85 eV range (i.e. with a time constant of 10 days), and that the detector had been cold for 50 days total, resulting in a $\sim e^4$ fold rate reduction compared to the final LS detector dataset (shown here). In CRESST detectors, both a slow ($\sim$ year) and a fast ($\sim$ 15 days) decay constant have been observed [4, 5]. We assume that the CRESST-III data was taken approximately one year into their run, when 6 fast time constants and 1 slow time constants had elapsed compared to the LS detector's final dataset, giving a $\sim e^7$ correction to the rate. In the right subfigure of Fig. S1 we make this

* These authors contributed equally to this work.; rkromani@gmail.com

correction, and see that rates in both the LS detector, the CPD detector, and the CRESST-III detector are broadly compatible. We hypothesize that all three detectors are limited by a background associated with the relaxation of mechanical stress in detector films, as described in Section 2.2 from the main text. Depending on the exact mechanism underlying this background, additional corrections (e.g. for aluminium film grain size) might explain any additional detector-to-detector rate variations.

SUPPLEMENTARY NOTE 2: ESTIMATING QUASIPARTICLE DENSITIES FOR REPRESENTATIVE QUANTUM SYSTEMS

Athermal phonons incident on a superconductor can break Cooper pairs in the superconductor, creating quasiparticles which can cause decoherence of superconducting qubits. The impact of "high energy" backgrounds on quantum circuit performance has recently been the subject of significant study [7–9]. In this section, we comment on the importance of such high-energy backgrounds relative to stress-induced backgrounds.

To this end, we separate the background observed in our calorimeters into two regions: the first between 3 and 85 eV (the two lowest-energy bins in Fig. 2 from the main text), and the second above 85 eV where the response of the detector saturates such that the energy deposited in the detector cannot be accurately reconstructed. We associate the first region with stress-related events (as argued in Section 2.2 from the main text) and the second region with cosmic-ray muons, high-energy gammas, neutrons, alphas, etc. interacting with the calorimeter. As we cannot accurately reconstruct the energy of these saturated events, we assume that each deposits 100 keV of energy in the phonon system, corresponding approximately to the energy deposited by a minimum ionizing muon [10] and similar to the average energy in Ref. [9].

In the simplest terms, we can compare the relative powers of the high-energy and stress-induced backgrounds. For example, in our first dataset, we see a rate of approximately 672 eV/s of stress events in the HS calorimeter, 10.9 eV/s in the LS calorimeter, and 5.8 keV/s of high-energy backgrounds in either calorimeter—summing the energies observed directly in the case of stress backgrounds and assuming 100 keV per saturated event in the case of high-energy backgrounds. In radiopure underground cryostats, as suggested in Ref. [8], the high-energy background power may be reduced by as much as a factor of 10^4, implying that stress-induced backgrounds may completely dominate the phonon-background power in such systems.

In many quantum circuits, the average quasiparticle density depends not only on the power of phonons incident on the superconducting elements but also on the average energy of the phonon events. Inspecting Eq. (2) in Sec 3.6 of the main text, we note that if the system is dominated by recombination (i.e., s is small, as in many quantum circuits [7]) and g is not constant with time, as is the case for both high-energy and stress-related quasiparticle creation, then the time-averaged quasiparticle density will in general be smaller for infrequent high-energy events than for frequent low-energy events, even if both backgrounds have similar powers. Dense quasiparticle populations created by infrequent high-energy events will recombine more quickly than more diffuse quasiparticle populations created by frequent low-energy events.

We simulated the time-dependent quasiparticle density in two cases: a recombination-limited superconductor modeled after Ref. [7] and a trapping-dominated superconductor based on Ref. [11] (as discussed in Sections 2.3 and 3.6 in the main text). In both cases, we simulate the quasiparticle densities based on measured phonon event energies and timing in our HS calorimeter, assigning a phonon energy of 100 keV for saturated events. Supplementary Figure 2 shows the simulated quasiparticle densities.

In the recombination-dominated case (i.e., insignificant quasiparticle trapping), we simulate a time-averaged value of $x_{qp} \approx 4.1 \times 10^{-8}$ due to stress events only and 5.0×10^{-8} if energy from high-energy backgrounds is included. The simulated reduced quasiparticle density due to high-energy backgrounds alone is 1.5×10^{-8}, which is comparable to the $x_{qp} \geq 7 \times 10^{-9}$ lower bound estimated in Ref. [7] for background radiation. While it is notable that the stress-induced and high-energy contributions to x_{qp} are comparable in this recombination-dominated case, this will not be generally true; stress-induced events may occur in different systems with significantly different rates or energy scales that depend on details of the experimental setup.

Multiple works [9, 12] have suggested using quasiparticle traps to suppress the time-averaged residual quasiparticle density. We simulate such a system (based on Ref. [11]) with the same event-rate assumptions as for the recombination-dominated case and estimate $x_{qp} = 3.6 \times 10^{-10}$ if high-energy backgrounds are included and 2.8×10^{-11} with only stress events included, approximately three orders of magnitude lower than measured in Ref. [11]. However, if we increase the average energy per stress event by a factor of 10^3, or equivalently increase the rate density of events by a factor of 10^3 and assume energy is much more locally absorbed, we recover $x_{qp} \approx 2.8 \times 10^{-8}$ both with and without high-energy backgrounds (as observed shortly after cooldown in Ref. [11]). This increased energy scale ($E \approx 10$ keV) is still lower than the scale of events observed by CRESST [13], suggesting that some combination of increases in rate and energy scale plausibly explains the time-varying quasiparticle density observed in Ref. [11].

Previously discussed mitigation techniques include operating radiopure quantum circuits in low-background environments [7, 8] (which would not reduce the stress-induced phonon background) and using phonon sinks to reduce the background athermal phonon populations [9]. Phonon sinks would reduce the effects of the stress-

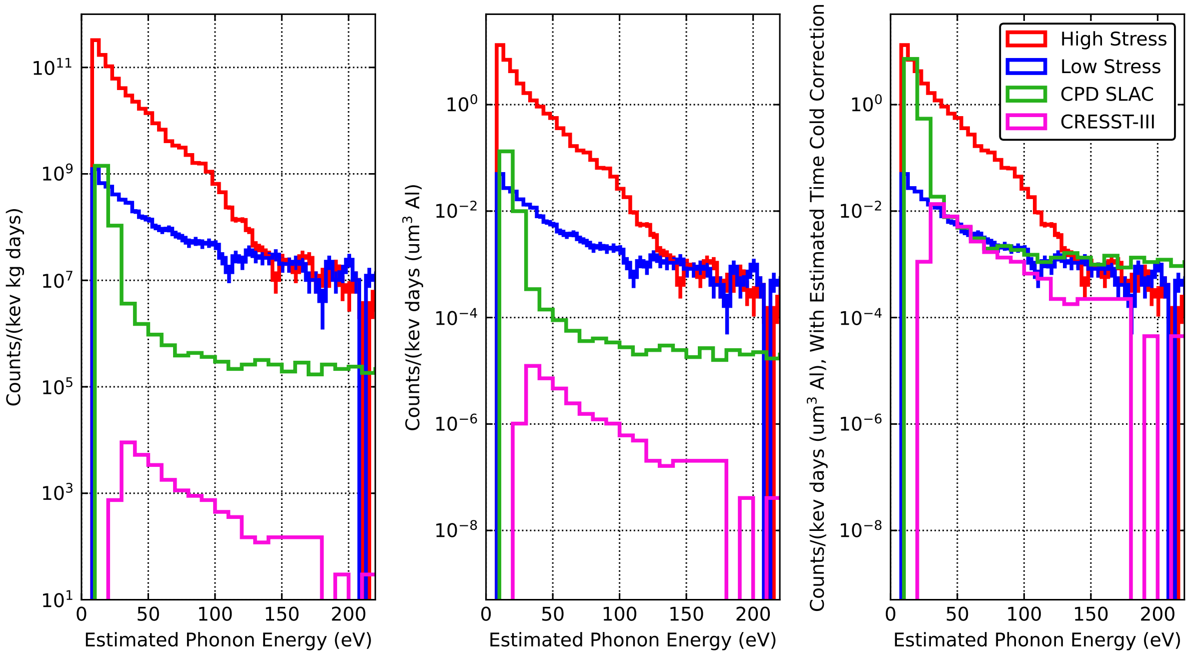

Supplementary Figure S1. **Comparison to CPD and CRESST.** Spectra of events in HS and LS calorimeters, compared to those measured with the CRESST-III detector [2] and by SuperCDMS in the CPD detector at SLAC [1]. Data are from the last of seven datasets taken. The energy in the HS and LS calorimeters are estimated assuming a phonon collection efficiency of 25%, and therefore differ by a constant factor from the spectra in Fig. 2 from the main text. The left subfigure normalizes by detector mass, the center subfigure normalizes by aluminium volume, and the right subfigure both normalizes by aluminium volume and applies an estimated time cold correction (see text in Supplementary Note 1). Error bars correspond to 1 sigma statistical errors for the LS and HS spectra; uncertainties could not be reconstructed for the CRESST or CPD detectors from the datasets included alongside Ref. [6]. Source data are provided as a Source Data file.

induced phonon background if the phonons are generated by, e.g., glue-crystal microfractures. However, phonon sinks would not significantly mitigate the effects of film-crystal microfractures, e.g., between the crystal and superconducting qubit thin films. In the latter case, energy would presumably be efficiently transferred to the quasiparticle system of the qubit without involving significant crystal-scale phonon propagation. As we have shown, trapping quasiparticles within the qubit [9, 12] would somewhat mitigate the effects of stress-induced athermal phonons, but a resolution of the underlying athermal phonon problem is needed to achieve thermal densities of quasiparticles.

In summary, our simulations indicate that in the case of the recombination-dominated qubit, stress and high-energy backgrounds plausibly contribute about equally to quasiparticle poisoning, while experimental evidence [11] indicates that the quasiparticle density is dominated by stress-induced quasiparticles in the trapping-dominated superconductor. In either case, the rate and energy scale of stress-induced events will presumably vary from experiment to experiment, either increasing or decreasing the relative importance of this background.

The simulated residual quasiparticle densities are summarized in Table S2, while the properties of the superconductors modeled in our simulations are listed in Table S3.

SUPPLEMENTARY NOTE 3: LOW STRESS TECHNIQUE DESCRIPTION

The low stress mounting technique described in this paper was developed over the course of multiple design iterations. As a general principle, the two most challenging aspects of this process were achieving high bond strengths and designing a setup such that the calorimeters could be bonded and transported without breaking the delicate and rigid structural bonds.

The bulk wire from which the structural bonds is constructed is in general far stronger than needed to mechanically support the gram scale detectors. However, both the bond itself and the interface between the bond and bonding surface are relatively weak. Optimizing this strength required a campaign of varying the e.g. bond force, bond time, etc. and pull testing a number of samples using a mechanical pull tester. Obtaining a bond which was both well bonded to the substrate and mechanically strong at the bond foot required settings in a relatively narrow range. Heuristically, we found that the strongest bonds we obtained failed at ∼0.3 the bulk bond breaking strength and would fail with either the bond pad being separated from the substrate, or from the bond foot snapping off of the bonding wire where it was deformed

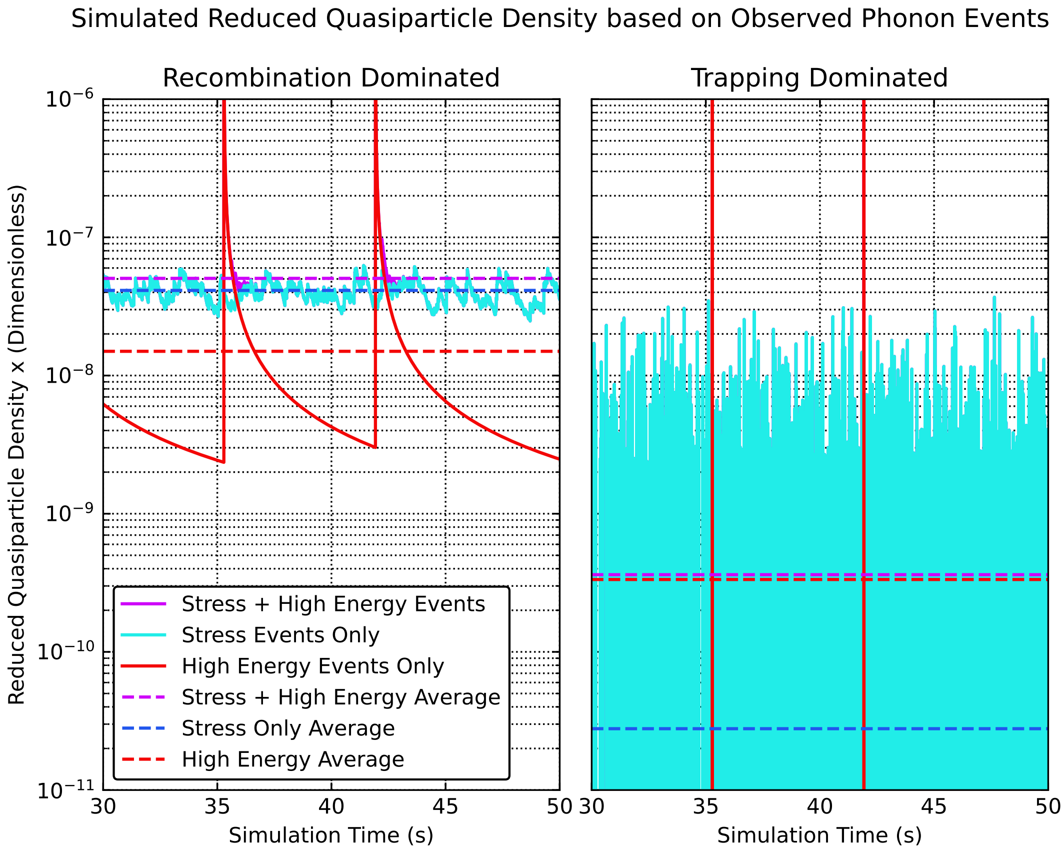

Supplementary Figure S2. **Simulated Reduced Quasiparticle Density x_{qp} in Recombination- and Trapping-Dominated Systems vs. Time.** Simulations are based on observed events in the high stress calorimeter (see Supplementary Note 2 for details). Note that in the trapping-dominated simulation (right), due to the fast trapping timescale both curves are only on scale for a short length of time after each event; the average value of x_{qp} is well below 10^{-9}. The recombination-dominated qubit was modeled after Ref. [7] and the trapping-dominating superconductor was modeled after Ref. [11]. Dotted lines are time averages. Source data are provided as a Source Data file.

at the "ankle." Structural bonds were made of 2 mil (50 μm) 1% Si aluminium wire from Tanaka, and were made on a manual WestBond wire bonding machine.

To support the devices during bonding, we used a 3D printed plastic jig, as shown in Fig. S3. This jig was designed to be highly rigid, such that force transferred to the calorimeter when bonds were made would not move the calorimeter enough to break bonds which were already made. The jig was constructed out of two pieces, the first that supported and centered the calorimeter being bonded from the bottom, and the second clamping the calorimeter to the bottom jig by pushing downwards. This top jig was designed to only contact the edge of the calorimeter to prevent damage to the films patterned on the surface, and was made relatively small to allow for access to the bonding pads around the device. The jig was also designed such that when removed, the calorimeter would not move relative to the copper support to which it was bonded. This ensured that the device would hang evenly without contacting support structures when the jig was removed.

The bonded device was transported to the cryostat while in the bonding jig to ensure that it would not be damaged during transport. The top and then bottom jigs were slowly removed, and the hanging device was mounted in the cryostat. In general, calorimeters could only be structurally bonded once due to bonding pad size constraints, so if the calorimeter was to be run again after completing a cryogenic run, it needed to be removed and stored without breaking the structural wire bonds. We found that it was easier to break these bonds when attempting to reinstall the bonding jig than it was to carefully transport and store the calorimeter in the hanging state. These suspended calorimeters were in general strong when accelerated side to side, but very weak when accelerated up and down. For example, the calorimeters would not be broken by moderate side to side manual shaking, but would easily be broken by even small ($\sim$mm) scale drops onto rigid surfaces.

During the initial development of this technique, we

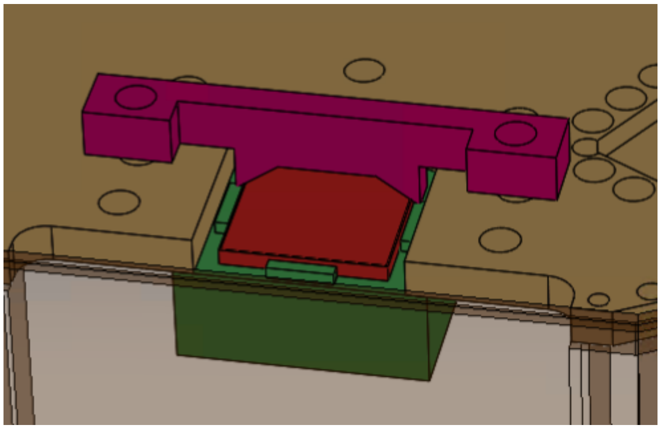

Supplementary Figure S3. **CAD drawing of the LS calorimeter bonding jig design.** Calorimeter (red) is held by 3D printed jigs (bottom, green; top, pink) while being bonded to the copper support plate (gold). An additional element of the housing (bottom half of image) was made semi-transparent to more easily show the jig configuration.

suffered from very poor ($\sim 10\%$) yield, but through iterative improvements to our design and techniques have obtained significantly improved ($\sim 80\%$) yields. Presumably additional engineering and process development could enable significantly higher ($\sim 95\%+$) yields.

TABLE S1. **High and Low Stress calorimeter rates and their time dependence.**

Stress, Bin	Relative Rate[a]	Decay Const. (1/d)	Fit τ (d)	χ^2/(dof)
High, 3–38 eV	1.000 ± 0.026	0.1659 ± 0.0039	6.03 ± 0.14	4192/68
High, 38–85 eV	$(4.93 \pm 0.58) \times 10^{-4}$	0.093 ± 0.023	10.8 ± 2.7	93.5/68
High, 85+ eV	$(4.83 \pm 0.20) \times 10^{-3}$	0.0017 ± 0.0070		90.7/68
Low, 3–38 eV	$(7.10 \pm 0.31) \times 10^{-3}$	0.1678 ± 0.0082	5.96 ± 0.29	124.8/68
Low, 38–85 eV	$(5.92 \pm 0.59) \times 10^{-4}$	0.100 ± 0.020	9.97 ± 1.96	83.1/68
Low, 85+ eV	$(4.96 \pm 0.19) \times 10^{-3}$	0.0076 ± 0.0062		70.2/68

Note: Uncertainties are 1σ. Fit τ is derived from the fit Decay Constant, and are not reported for the high energy bins for either calorimeter, as the fit decay constants are consistent with being flat with respect to time

[a] Fit rate relative to the fit rate in the high stress 3–38 eV bin (60 hours after cooldown).

TABLE S2. **Quasiparticle Background Simulation Results.**

	Average Power	Recombination Dominated x_{qp} [a]	Trapping Dominated x_{qp} [b]
High-energy Backgrounds	5.8 keV/s	1.5×10^{-8}	3.3×10^{-10}
Stress-induced Backgrounds	672 eV/s	4.1×10^{-8}	2.7×10^{-11}
Stress + High Energy	6.5 keV/s	5.0×10^{-8}	3.6×10^{-10}

[a] Modeled after a recombination-dominated qubit in Ref. [7].

[b] Modeled after a trapping-dominated superconductor in Ref. [11].

Modeled x_{qp} and measured powers derived from HS dataset 1.

TABLE S3. **Superconductor Properties used in Residual Quasiparticle Density Simulations.**

	Recombination-Dominated Superconductor [a]	Trapping-Dominated Superconductor [b]
Thickness	200 nm	35 nm
Superconductor Coverage Fraction	100 %	40%
Recombination Time Constant r	$(20\ \text{ns})^{-1}$	$(20\ \text{ns})^{-1}$
Trapping Rate s	0 Hz	8 kHz
Phonon Collection Efficiency	50%	50%

[a] Modeled after a recombination-dominated qubit in Ref. [7].

[b] Modeled after a trapping-dominated superconductor in Ref. [11].

TABLE S4. **Table of datasets taken.**

Dataset Number	Start Date	Start Time (UTC)	Length (Hours)
1	2022-05-03	21:54:54	12.06
2	2022-05-04	18:15:33	12.06
3	2022-05-05	11:23:22	10.06
4	2022-05-05	22:25:41	10.06
5	2022-05-06	10:08:08	12.07
6	2022-05-06	22:58:07	12.07
7	2022-05-07	22:32:50	12.07

TABLE S5. Summary of the passage fraction of randomly selected events in each of the 7 data taking periods, showing passage fraction for the baseline, slope, and χ^2 cuts. These events do not necessarily contain a pulse. These randomly selected events allow for us to study the passage fraction of infinitesimally small events.

Dataset No., Stress State	Baseline	Slope (Sequential)	Slope (Total)	χ^2 (Sequential)	χ^2 (Total)	Total Passage
1, Low Stress	0.964	1.00	0.964	0.973	0.973	0.939
2, Low Stress	0.966	1.00	0.966	0.984	0.984	0.951
3, Low Stress	0.962	1.00	0.965	0.995	0.995	0.960
4, Low Stress	0.969	1.00	0.969	0.981	0.981	0.951
5, Low Stress	0.965	1.00	0.965	0.979	0.978	0.944
6, Low Stress	0.970	1.00	0.970	0.985	0.985	0.956
7, Low Stress	0.968	1.00	0.969	0.983	0.983	0.951
1, High Stress	0.952	1.00	0.952	0.970	0.970	0.923
2, High Stress	0.952	1.00	0.946	0.977	0.977	0.930
3, High Stress	0.940	1.00	0.956	0.970	0.970	0.927
4, High Stress	0.959	1.00	0.959	0.937	0.937	0.898
5, High Stress	0.959	1.00	0.959	0.978	0.977	0.937
6, High Stress	0.960	1.00	0.960	0.979	0.979	0.939
7, High Stress	0.959	1.00	0.963	0.982	0.982	0.936
1, High + Low Stress						0.869
2, High + Low Stress						0.889
3, High + Low Stress						0.892
4, High + Low Stress						0.857
5, High + Low Stress						0.888
6, High + Low Stress						0.901
7, High + Low Stress						0.893

Cuts are applied sequentially. Sequential passage is the fraction of events that passed the previous cut which also pass this cut, total passage is the fraction of all events that pass this cut.
High + Low Stress total passage fractions show the passage fraction for the combined total high and low stress cuts.

TABLE S6. Summary of the passage fraction of triggered events in each of the 7 data taking periods, showing passage fraction for the baseline, slope, and χ^2 cuts.

Dataset No., Stress State	Baseline	Slope (Sequential)	Slope (Total)	χ^2 (Sequential)	χ^2 (Total)	Total Passage
1, Low Stress	0.959	0.976	0.965	0.973	0.973	0.921
2, Low Stress	0.964	0.977	0.971	0.981	0.981	0.935
3, Low Stress	0.964	0.974	0.969	0.990	0.990	0.942
4, Low Stress	0.967	0.976	0.972	0.980	0.980	0.935
5, Low Stress	0.963	0.974	0.969	0.975	0.975	0.927
6, Low Stress	0.969	0.977	0.974	0.981	0.981	0.939
7, Low Stress	0.969	0.977	0.973	0.979	0.979	0.936
1, High Stress	0.931	0.960	0.940	0.961	0.961	0.868
2, High Stress	0.935	0.961	0.944	0.966	0.966	0.877
3, High Stress	0.951	0.964	0.946	0.964	0.964	0.881
4, High Stress	0.939	0.964	0.946	0.925	0.925	0.846
5, High Stress	0.939	0.964	0.948	0.963	0.963	0.881
6, High Stress	0.940	0.963	0.950	0.968	0.968	0.887
7, High Stress	0.946	0.966	0.954	0.978	0.978	0.894
1, High + Low Stress						0.803
2, High + Low Stress						0.826
3, High + Low Stress						0.834
4, High + Low Stress						0.796
5, High + Low Stress						0.821
6, High + Low Stress						0.838
7, High + Low Stress						0.843

Cuts are applied sequentially. Sequential passage is the fraction of events that passed the previous cut which also pass this cut, total passage is the fraction of all events that pass this cut.
High + Low Stress total passage fractions show the passage fraction for the combined total high and low stress cuts.

REFERENCES

[1] I. Alkhatib *et al.* (SuperCDMS), Light Dark Matter Search with a High-Resolution Athermal Phonon Detector Operated Above Ground, Phys. Rev. Lett. **127**, 061801 (2021), arXiv:2007.14289 [hep-ex].

[2] A. H. Abdelhameed *et al.* (CRESST), First results from the CRESST-III low-mass dark matter program, Phys. Rev. D **100**, 102002 (2019), arXiv:1904.00498 [astro-ph.CO].

[3] R. Strauss *et al.* (CRESST), A prototype detector for the CRESST-III low-mass dark matter search, Nucl. Instrum. Meth. A **845**, 414 (2017), arXiv:1802.08639 [astro-ph.IM].

[4] G. Angloher *et al.*, Latest observations on the low energy excess in CRESST-III, SciPost Phys. Proc. **12**, 013 (2023), arXiv:2207.09375 [astro-ph.CO].

[5] D. Fuchs, The low energy excess in CRESST-III (2023), EXCESS2023 Workshop.

[6] P. Adari *et al.*, EXCESS workshop: Descriptions of rising low-energy spectra, SciPost Phys. Proc. **9**, 001 (2022), arXiv:2202.05097 [astro-ph.IM].

[7] A. Vepsäläinen *et al.*, Impact of ionizing radiation on superconducting qubit coherence, Nature **584**, 551 (2020), arXiv:2001.09190 [quant-ph].

[8] L. Cardani *et al.*, Reducing the impact of radioactivity on quantum circuits in a deep-underground facility, Nat. Commun. **12**, 2733 (2021), arXiv:2005.02286 [cond-mat.supr-con].

[9] J. M. Martinis, Saving superconducting quantum processors from decay and correlated errors generated by gamma and cosmic rays, npj Quantum Inf. **7**, 90 (2021), arXiv:2012.06137 [quant-ph].

[10] R. L. Workman *et al.* (Particle Data Group), Review of Particle Physics, Prog. Theor. Exp. Phys. **2022**, 083C01 (2022).

[11] E. T. Mannila, P. Samuelsson, S. Simbierowicz, J. T. Peltonen, V. Vesterinen, L. Grönberg, J. Hassel, V. F. Maisi, and J. P. Pekola, A superconductor free of quasiparticles for seconds, Nat. Phys. **18**, 145 (2022), arXiv:2102.00484 [cond-mat.supr-con].

[12] R.-P. Riwar, A. Hosseinkhani, L. D. Burkhart, Y. Y. Gao, R. J. Schoelkopf, L. I. Glazman, and G. Catelani, Normal-metal quasiparticle traps for superconducting qubits, Phys. Rev. B **94**, 104516 (2016), arXiv:1606.04591 [cond-mat.supr-con].

[13] J. Åström *et al.*, Fracture Processes Observed with A Cryogenic Detector, Phys. Lett. A **356**, 262 (2006), arXiv:physics/0504151.
